# Lopinavir/Ritonavir Impairs Physical Strength in Association with Reduced Igf1 Expression in Skeletal Muscle of Older Mice

**DOI:** 10.4172/2155-6113.1000216

**Published:** 2013-06-25

**Authors:** Siu Wong, Shalender Bhasin, Carlo Serra, Yanan Yu, Lynn Deng, Wen Guo

**Affiliations:** Research Program in Men’s Health: Aging and Metabolism, Boston Claude D. Pepper Older Americans Independence Center for Function promoting Anabolic Therapies, Brigham and Women’s Hospital, Harvard Medical School, Boston, USA

**Keywords:** HIV protease inhibitor, Age and overweight, Insulin resistance, Insulin-like growth factor 1, Muscle, Adipose tissue, Physical strength and mobility

## Abstract

**Background:**

Late-middle age HIV patients are prone to fatigue despite effective viral control by antiretroviral therapies. Rodent models to recapitulate this phenotype are still not available.

**Hypothesis:**

Drug treatment may compromise muscle strength and physical performance more in older individuals with pre-existing metabolic disorders than normal young ones.

**Methods:**

Kaletra was given to overweight male mice at late-middle age and normal young adults; both on a rodent diet containing 30% fat calorie. Body composition and grip strength were measured at baseline and after drug treatment. Rota-rod running, insulin and glucose tolerance were measured at the end of the experiment. Drug effect on metabolic activity and spontaneous movements were assessed using the metabolic cage system. Representative muscle and fat tissue were analyzed for protein and mRNA expression. Selected findings were tested using murine C2C12 myotubes.

**Results:**

Kaletra reduced grip strength in both young and older mice but impaired rotarod performance only in the old. Spontaneous movements were also reduced in Kaletra-treated old mice. Kaletra reduced IGF-1 expression in all muscle groups tested for the old and in cultured myotubes but to a less extent in the muscle of young animals. Reduced IGF-1 expression correlated with increased expression of muscle-specific atrogene MAFbx and MuRF1. Kaletra also increased abdominal fat mass markedly in the old animals and to a less extend in the young.

**Conclusion:**

Long-term Kaletra intake aggravated abdominal obesity and impaired muscle strength. This effect was worse in older animals than in normal young adults.

## Introduction

Successful antiretroviral therapy significantly increases mortality and increasing attention is paid to associated morbidities, such as complaints of weakness and fatigue, which are common complaints in patients chronically taking antiretroviral therapy [1–3]. Both viral infection and antiretroviral drugs may contribute to this syndrome. Since it is not feasible to study long-term drug side-effect on non-infected humans, reproduction of clinical side effects in a rodent model can be helpful to facilitate research in this area. Indeed, drug-related insulin resistance, dyslipidemia, and mitochondrial toxicity have been reported in rodents treated with antiretrovirals [4–12]. Limitations for these studies include exclusive use of healthy young animals and paucity of functional measurements. Since most HIV patients in USA are now ≥ 50 years (www.cdc.gov/hiv), over-weighted, and with fatty diet, outcomes of studies on young rodents may not reflect the drug effects experienced by these real-life patients. For instance, Kaletra markedly increases plasma lipids in healthy volunteer [13], which was recapitulated in young mice [9]. However, HIV patients taking Kaletra display only modest increase in plasma lipids [14,15]. In this work, we investigated the effect of Kaletra on over-weighted mice at a late-middle age with a high fat diet, conditions that are more reflective of the reallife patient conditions. For comparison, a cohort of young adult mice was studied in parallel. Kaletra (lopinavir/ritonavir 4:1) was selected for this work because of its proven long-term efficacy [16].

## Material and Methods

This study aims to determine the effect of Kaletra on functional and metabolic health in older mice with pre-existing over-weighted. Selected experiments were conducted in young mice for comparison. This research complied with the NIH guidelines on laboratory animal care and Institutional Animal Care and Use Committees (IACUC) of Boston University approved the protocol. Mice of two age groups were used. The older ones were at 20 month and over-weighted (~30% body fat mass at baseline) and the younger ones were at 6 month (15% body fat). Both were given a high fat food of 30% fat calorie. Detailed methods on diet, drug administration, and serum drug measurement, functional and molecular measurements are presented in the supplemental information section.

## Results

### Effects of kaletra on physical performance and body composition in older mice with pre-existing overweight

**Figure 1:**
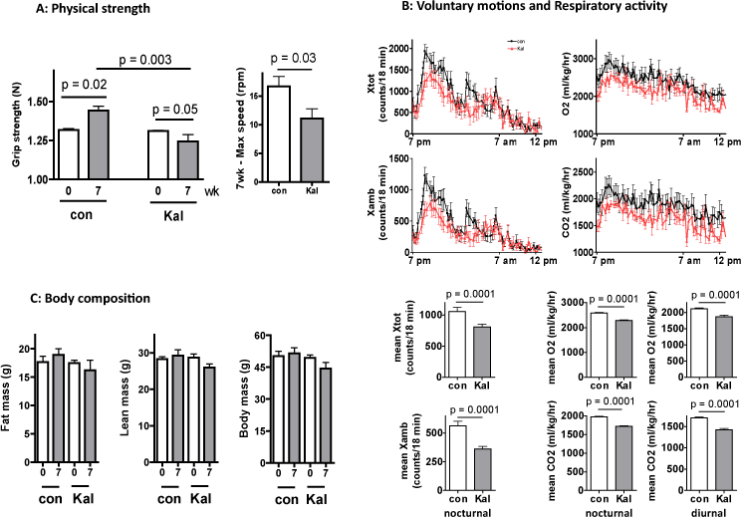
Long-term treatment with Kaletra impairs physical strength and reduces spontaneous physical and metabolic activity without gross change of body composition A. Grip strength (Left panel) measurement by an automatic force transducer (Columbus Instruments, Columbus, OH). Max speed (right panel) of physical performance measured by Rota Rod (Columbus Instruments, Columbus, OH). B. Activity (Left panel), oxygen (O2) consumption (upper right), and carbon dioxide (CO2) production were measured by Oxymax system (Columbus Instruments, Columbus, OH). C. Body composition determined by nuclear magnetic resonance (NMR; EchoMRI-100, Echo Medical System, Houston, TX). Kaletra was provided to C57BL/6 mice beginning at age 20 month when the mice were moderately overweight. The drug was mixed with food (0.1 % wt/wt) for form pellets using a commercial service. The same diet without drug was given to the control animals. (**A**, left panel) Grip strength was measured at baseline and in week 7 after drug treatment. (**A**, right panel) Maxium speed of rota-rod running achieved by the animals in week 7. (**B**, upper panel) Spontaneous movements, O_2_ consumption, and CO_2_ production rate recorded in the metabolic cages between 7 pm and 12 am (darker period 7 pm – 7 am), measured in week 7. Xtot and Xamb: total movements and ambulatory movements, both recorded as the number of beam breaking along the X-direction. (**B**, lower panel) Mean values of parameters measured above during nocturnal and diurnal periods. (**C**) Body mass at baseline and in week 7, measured by in vivo NMR. Results are shown as means +/− se, N = 6 for each group. Between-group comparison was analyzed by Student’s *t* test.

Animals were first assessed by their grip strength before and after seven weeks of Kaletra treatment. As shown in Figure 1A (left panel), the control group displayed an increase of grip strength after the dietary switch, while Kaletra-treated mice showed a decrease in grip strength in the same period, resulting in a significant 14% difference between the two groups. The maximal speed that Kaletra-treated mice could reach on a rota-rod was also substantially lower than that of the control animals (Figure 1A, right panel). Using the metabolic cage system, we found that Kaletra reduced the nocturnal activity (Figure 1B, left panel) but not diurnal activity (data not shown). O2 consumption and CO2 production were reduced in the Kaletra-treated animals in both nocturnal and diurnal periods (Figure 1B, right panel). Kaletra (data not shown) did not affect respiration exchange rate. Despite these changes in physical activity and metabolic rate, we found no gross drug effect on body weight and composition measured by *in vivo* NMR (Figure 1C).

### Effect of kaletra on plasma lipids, liver, fat, and muscle mass

**Figure 2:**
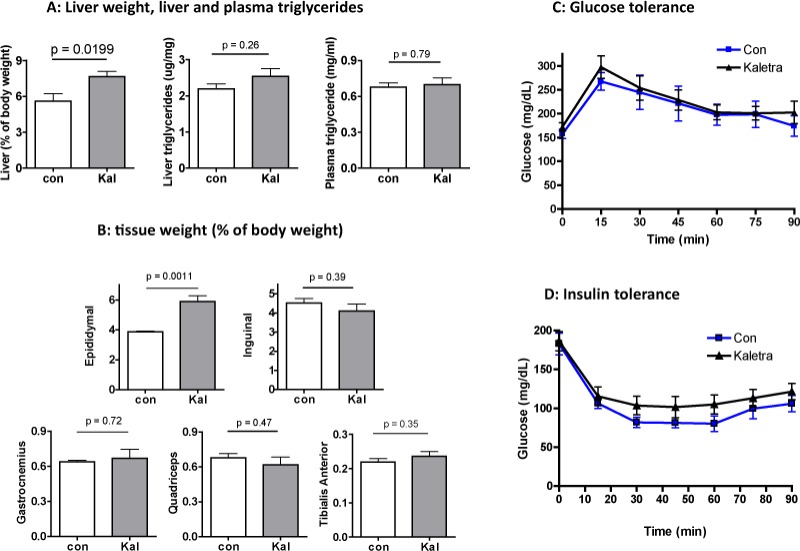
Effects of Kaletra on body composition, plasma and hepatic lipids, insulin and glucose tolerance (**A**) Liver weight, liver triglyceride concentration and plasma triglyceride concentration. (**B**). Tissue weight for major fat depots and hind limb muscle groups, expressed as percentage of body weight. (**C**) Glucose tolerance test. (**D**) Insulin tolerance test. Results are shown as means +/− se, N = 6 for each group. Between-group comparison was analyzed by Student‘s *t* test.

As shown in Figure 2A, liver weight was increased in mice treated with Kaletra but liver or plasma triglyceride concentrations were similar between the two groups, which were somewhat unexpected based on previous rodent studies [8–11], but nevertheless compatible with clinical reports that over 90% of the patients taking Kaletra don’t show dramatic increase in plasma lipids (< 12%) [14,15]. Kaletra has been shown to strikingly reduce fat mass in young mice [9,11]. However, in the old over-weighted mice used for this work, Kaleta caused a 40% increase in abdominal epididymal fat, without affecting inguinal fat (Figure 2B, upper panel). A decrease in perirenal fat mass was found (Figure s1), similar to that reported by others [11]. As shown in Figure 2B (lower panel), despite a decrease in grip strength and spontaneous activity, we were not able to detect any difference in muscle weight for gastrocnemius and quadriceps, two major weight-bearing muscles with different ratio of fast- and slow-twitching fibers, and tibialis anterior (TA) which consists of primarily fast-twitching myofibers. No significant difference was found in muscle fiber cross-sectional areas either after histological staining (data not shown). These results are in agreement with the clinical reports showing no correlation between lean mass and frailty in HIV patients taking anti-retrovirals [17].

### Effects of kaletra on insulin and glucose tolerance

Kaletra and other protease inhibitors can cause acute but reversible inhibition on insulin-stimulated glucose uptake [4]. To assess the long-term drug effect on insulin resistance, we performed insulin and glucose tolerance tests on the animals. Both control and Kaletra-treated animals has similar fasting (150–165 mg/dl) and non-fasting (175–180 mg/dl) glucose concentrations. Kaletra did not affect glucose tolerance (Figure 2C) nor insulin tolerance (Figure 2D). Although the blood glucose trough appeared moderately higher in the Kaletra-treated animals (Figure 2D), the calculated AUC (area under curve) between 15–90 min was not significant different (9190 +/− 278 vs 10510 +/− 546 for control and Kaletra groups, respectively, means +/− se, p=0.4, n=6). No difference was detected in serum insulin concentration either (0.55 +/− 0.05 vs. 0.49 +/− 0.04 ng/ml for control and Kaletra-treated groups, p=0.4, n= 6). These results are also compatible with the relevant clinical observations [15,18].

### Kaletra does not reduce fat tissue expression of markers for adipogenesis

**Figure 3:**
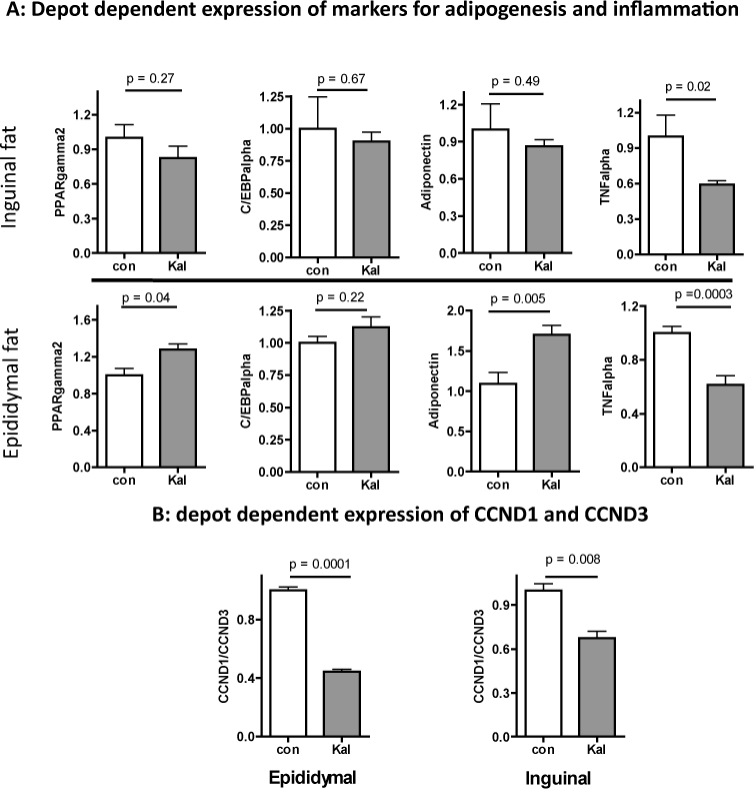
Effects of Kaletra on fat tissue mRNA expression (**A**) Expression of adipocyte transcription factor PPARγ2 and C/EBPα, adiponectin, and TNFalpha in abdominal epididymal (upper panel) and subcutaneous inguinal (lower panel) fat depots. (**B**) The ratio of mRNA expression between cyclin D1 and cyclin D3 (CCND1/CCND3). All results are normalized to house-keeping gene HPRT, shown as means +/− se. N = 6, Student’s *t* test.

Lopinavir may inhibit adipocyte differentiation *in vitro* but the *in vivo* results are inconclusive [19–22,8,9]. As shown in Figure 3A (upper panel), Kaletra did not change inguinal fat expression of (Peroxisome proliferator-activated receptor gamma) PPARγ2 and Ccaat-enhancer-binding proteins (C/EBPα), both are master transcription factors that coordinately regulate adipogenesis [23] or their downstream target adiponectin but reduced expression of inflammatory cytokine TNFα, a cytokine known to inhibit adipogenesis [24]. In epididymal fat, Kaletra moderately increased expression of PPARγ2 and adiponectin and simultaneously reduced expression of TNFα (Figure 3A, lower panel). Together, these results do not suggest Kaletra negatively regulate adipocyte differentiation *in vivo*. Additionally, Kaletra reduced the ratio of cyclin D1 (CCND1) to cyclin D3 (CCND3) in both inguinal and epididymal fat (Figure 3B). Since D1 is typically found in proliferating cells while D3 is important for fat cell terminal differentiation and maintenance [25–27], a reduction in the D1/D3 ratio suggests that Kaletra promote fat cell differentiation *in vivo*, which could contribute to explain the marked increase of epididymal fat mass (Figure 2B). Besides, Kaletra-associated reduction in TNFα expression implies that fat tissue inflammation is unlikely a main contributor to the drug-induced loss of *in vivo* physical and metabolic function.

### Kaletra suppresses muscle expression of local IGF1 (IGF-1Ea), reduces mTOR activity, and reciprocally increases expression of muscle-specific atrogenes

**Figure 4:**
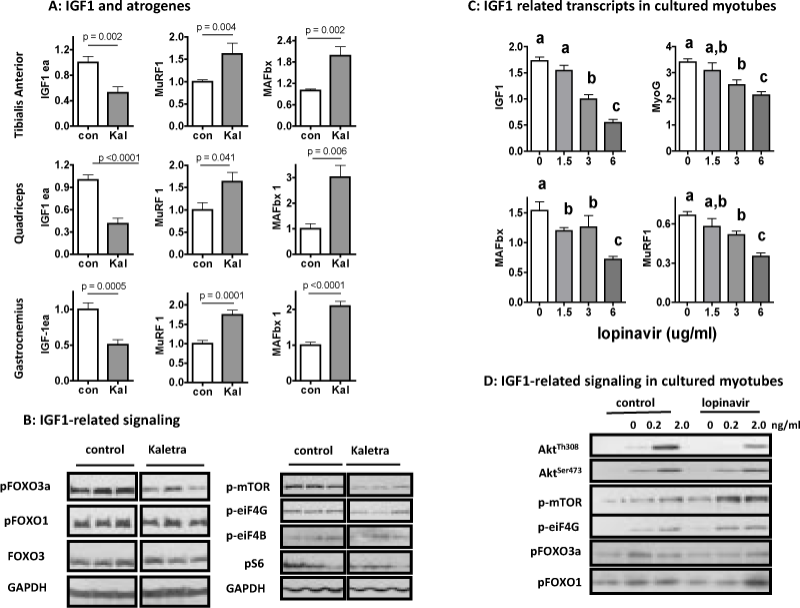
Effects of Kaletra on muscle IGF1-related molecular changes in skeletal muscle and cultured myotubes (**A**) Messenger RNA expression of IGF-1Ea and related muscle atrogenes in tibialis anterior (TA), quadriceps (Quad) and gastrocnemius (Gastro) muscle groups. Expression of each gene was first normalized to house-keeping gene HPRT and presented as a fraction of the control. (**B**, left panel) Western analysis of phosphor-Foxo3a^ser318/321^, phosphor-Foxo1^ser256^, and total Foxo3a in quadriceps muscle, with GAPDH as loading control. (**B**, right panel) Western analysis for phosphor-mTOR^ser2448^, phosphor-eiF4G^ser1108^, phosphor-eiF4B^ser422^, and phosphor-S6^ser235/236^ in quadriceps, with GAPDH as loading control. Results are representative of N = 6 for each group with three of each group for display. (**C**) Expression of IGF1, myogenin (MyoG), MAFbx and MuRF1 in cultured C2C12 myotubes exposed to different concentration of lopinavir (lopinavir:ritonavir = 4:1). The experiment was repeated twice in duplicates. Results were normalized to house-keeping gene HPRT and shown as means+/− se, a > b > c, p < 0.05, analyzed by One-way ANOVA with post hoc Tukey’s test. (**D**) Western analysis of C2C12 myotubes treated with low and high dose of IGF1 for the phosphorylation of relevant downstream targets. Myotubes were pre-incubated with lopinavir/ritonavir (4:1) at 3 ug/ml of lopinavir in 2% horse serum for 24 h. Cells were then washed with serum-free medium and incubated with serum-free medium added the same concentration of lopinavir/ritonavir for two hours. IGF1 was then added and cells were harvested after 30 mins. Results are representative of three independent experiments.

Insulin-like growth factor IGF-1Ea is a locally produced autocrine/paracrine that promotes muscle repair and regeneration while suppressing catabolism, which also contributes to muscle strength [28–30]. As shown in Figure 4A, IGF-1Ea was down regulated by Kaletra in all three muscle groups tested, with a reciprocal correlation with expression of MuRF1 and MAFbx, two of the muscle-specific E3 ubiquitin ligases typically up-regulated in acute muscle atrophy [28]. Western analysis shows that Kaletra reduced phosphorylation (inactivated form) of Foxo3a and, to a less extent, Foxo1, the downstream target of IGF1 and also the transcription factors of MuRF1 and MAFbx. As shown in Figure 4B (right panel), loss of muscle IGF-1Ea was associated with reduced phosphorylation (activated form) of mTOR, the master anabolic regulator, and its downstream target eiF4G, implying that long-term treatment with Kaletra negatively regulates protein translation in muscle. However, Kaletra equally inhibited not every component in this pathway. For instance, Kaletra (Figure 4B) did not reduce phosphorylation (activated form) of eiF4B and S6. The discordance may suggest activation of endogenous compensatory mechanism at various levels trying to maintain protein translation, which might explain the relatively well-maintained muscle mass (Figure 2B).

### Effect of Kaletra on young adult mice

The results described thus far suggest that long-term Kaletra intake reduces skeletal muscle strength in the over-weighted old mice. We also tested whether this effect occurred in drug-treated young adults. As shown in supplemental Figure s2, young mice displayed a marked increase in body weight after diet switching which was primarily attributed to fat mass increment. The control also showed a small increase in lean mass which was not observed in Kaletra-treated ones (Figure s2, upper right panel). Kaletra did not affect subcutaneous and increased epididymal fat only marginally (Figure s2). The drug increased liver weight but did not change liver or serum triglyceride concentrations (Figure s2) and had no effect on skeletal muscle mass for all three muscle groups examined (Figure s2). The younger mice had greater grip strength at baseline than the older ones (1.46 +/− 0.05 N vs. 1.32 +/− 0.03 N, for the young and the old, respectively, p=0.0001, n=12 for the old and n=20 for the young). After drug treatment, grip strength did not change in the control but was reduced in the Kaletra-treated group (Figure s3), as was found in the older ones (Figure 1A). However, Kaletra did not affect the rota-rod performance in the young (both control and Kaletra-treated mice were able to run beyond 35 rpm for > 30 min without falling, which was more than twice of the speed achieved by the old control). This result is in agreement with a recent report by Pistell et al showing that Kaletra impairs cognitive performance but not motor performance in adult mice [11]. Since grip strength positively correlates with cognitive function [31–35], Kaletra-induced decline in grip strength could be partially related to cognitive impairment reported by Pistell et al. [11].

Consistent with the data on the older animals, Kaletra had no effect on glucose tolerance in the young (Figure s4A). Kaletra did not affect insulin-stimulated rapid drop of blood glucose but delayed the recovery (Figure s4B) with a smaller area under curve in the insulin tolerance test than the control with AUC of 14790 +/− 827 for the control and 12180 +/− 591 for the Kaletra group (arbitrary unit, p=0.017, n= 10). Consistently, serum insulin concentration was lower in Kaletra-treated younger mice (Figure s4C). These results are consistent previous studies showing that selected HIV protease inhibitors can reduce serum insulin in mice under both low fat and high fat dietary conditions [10], although opposite findings have also been reported [11].

As shown in Figure s5, similar to the results from older mice, Kaletra markedly reduced IGF-1Ea expression in TA muscle in young mice, associated with increased expression of its downstream gene MuRF1 but MAFbx was not affected. Kaletra also reduced expression of IGF-1Ea in gastrocnemius muscle although to a less extent and with no significant impact on expression of MuRF1 or MAFbx. For quadriceps, Kaletra-related reduction in IGF-1Ea expression was marginal but an increase in MuRF1 mRNA was detected, with no effect on MAFbx. These results, together with the lack of drug effect on rotarod performance, suggest that young mice could be less vulnerable to Kaletra-mediated impairment on skeletal muscle strength than the old ones.

### Kaletra inhibits IGF1 expression and signaling in culturally differentiated murine myotubes

To test whether Kaletra-induced impairment on skeletal muscle strength could be a result of direct interaction between the drug and muscle cells, we studied the effect of lopinavir and ritonavir on cultured murine C2C12 myotubes. We were able to recapitulate some but not all of the *in vivo* effects of Kaletra on muscle. Incubation with lopinavir/ritonavir (4:1) for 24 h resulted in a dose-dependent reduction in IGF1 expression in differentiated myotubes (Figure 4C) and undifferentiated myoblasts (Figure s6). Myogenin, the transcription factor that coordinates skeletal muscle development and repair, was dose-dependently inhibited by lopinavir/ritonavir, implying that Kaletra had a direct inhibitory effect on myotube development. In addition, lopinavir/ritonavir reduced phosphorylation of Foxo3a/Foxo1 as well as its upstream Akt^Th308^ when myotubes were incubated in a basal medium without or with exogenous IGF-1 at a low concentration (Figure 4D). This effect was reversed by high concentration of exogenous IGF1. However, in contrast to the *in vivo* findings, lopinavir/ritonavir did not reduce but enhanced basal and IGF1-induced activation of mTOR and its downstream eiF4G (Figure 4D). Furthermore, drug-induced loss of IGF1 and activation of Foxo3a/Foxo1 in cultured myotubes were not correlated with an increase in expression of MuRF1 and MAFbx (Figure 4C). This is probably because both MuRF1 and MAFbx are muscle-specific proteasome ligases whose expression is positively correlated with the progression of myotube development. However, even when normalized to the expression level of myogenin, lopinavir/ritonavir still did not correlate with MuRF1/MAFbx expression (not shown). Together, these results demonstrated that alternative signaling pathways are involved in regulation of MuRF1/MAFbx expression under cell culture condition.

## Discussion

We reported the first evidence that long-term treatment with Kaletra in old male over-weighted mice resulted in a decrease in grip strength, rota-rod performance, spontaneous activity and metabolic activity. Similar decrease in grip strength was found in Kaletra-treated young animals but with no effect on rota-rod performance, (metabolic cage was not used for the young). No drug effect was detected on muscle mass in both age groups. The drug-induced functional impairments could reflect compromised muscle strength and cognitive decline [36]. Our findings are in line with the clinical observations that suggest a disconnection between steady muscle mass and compromised muscle strength in HIV patients taking antiretroviral therapies [37–40].

In association with a loss of gross physical performance, we reported the first evidence that Kaletra reduced local IGF1 expression in skeletal muscle. This effect was found to be more consistent in the old mice among three muscle groups with different myofiber composition and weight-bearing capacity, whereas the results in the young mice were less conclusive. The finding was recapitulated in cultured myotubes and myoblasts, implying a result of direct interaction between the drug and muscle cells. The drug-induced loss of IGF 1 was in turn associated with reduced suppression of Foxo1/3a both *in vivo* and *in vitro*. Kaletra was associated with a loss in activation of mTOR/eiF4G and increased expression of muscle-specific atrogenes MuRF1 and MAFbx. Similarly correlated reciprocal change between IGF1 and MuRF1/MAFbx has been reported in skeletal muscle atrophy of different causes [28]. The drug-induced loss of IGF1 could be responsible for the loss of suppression on Foxo1/3a [41,42], which in turn increases transcription of MuRF1/MAFbx [43–45]. It has been shown that infusion of indinavir increases muscle expression of MuRF1 without changing systemic IGF1 concentration but the drug effect on local IGF1 was not analyzed [46]. Of note, although widely used as a marker for muscle atrophy, the actual role of MuRF1/MAFbx remains a subject of debate [47]. Hence, they may serve as molecular markers for drug-mediated change in skeletal muscle but not necessarily the cause for the functional decline in these animals.

Another important finding from this work is that Kaletra increased abdominal fat in old mice that were already overweight but less so in young mice. This is in line with the commonly documented abdominal obesity in older HIV patients taking antiretroviral drugs [17]. Our data, to our knowledge, documented the first such observation in a rodent model as previous studies exclusively show that antiretroviral drugs reduce body weight in rodents [9,11,48]. The discrepancy may be related to age and body composition of the animals at the baseline and suggests that older animals with pre-existing overweight may be a better model for this type of studies. Additionally, the approaches of drug delivery may contribute to the different outcomes between the current and previous studies (see supplemental information). Consistent with the increase of fat mass, our data suggest that long-term treatment with Kaletra do not inhibit adipocyte differentiation at least in epididymal fat depot, as evidenced by increased expression of PPARgamma2 and adiponectin, decreased expression of cytokine TNFalpha and decreased ratio of CCND1/CCND3. Although in contrast with previous *in vitro* studies [19,49,50], the discrepancy is not unexpected considering the high drug doses commonly used in cell culture studies.

In summary, our data demonstrated that long-term treatment with Kaletra caused marked impairment of muscle strength measured as grip strength, rota-rod performance, and nocturnal spontaneous movements and metabolic activity in old weighted mice. The drug caused a similar impairment of grip strength in young adult animals but not in rota-rod running. IGF1 expression was reduced in all muscle groups examined for the old mice and in some but not as conclusive for the young. Reduced IGF1 expression was associated with an increase in expression of muscle atrogenes. The drug-induced inhibition on IGF1 expression was recapitulated in cultured myotubes. Our data demonstrated that Kaletra impaired muscle strength at both a functional level and a molecular level, causing more damage in the old over-weighted animals than in the young. These drug side effects occurred without gross loss of muscle mass. Based on the well-established role of IGF1 in muscle development and maintenance, as well as its proven capacity to reverse muscle atrophy in a variety of patho-physiological conditions [51,52], it will be interesting to investigate if the Kaletra-induced functional decline can be offset by pharmaceutical manipulation of muscle IGF1 signaling.

## Supplementary Material

Supplementary files
