# Supplementary material for: Lopinavir/Ritonavir Impairs Physical Strength in Association with Reduced Igf1 Expression in Skeletal Muscle of Older Mice
Source: J AIDS Clin Res. Author manuscript; Available in PMC 2015 Aug 4. (PMC4524660; doi:10.4172/2155-6113.1000216)
Supplement: Supplementary files [file NIHMS516715-supplement-Supplementary_files.zip › supplemental information.doc]

**Supplemental 1**

1. **Material and Methods**
   1.1. **Animals**

This research complied with the NIH guidelines on laboratory animal care and the protocol was approved by IACUC of Boston University. Male C57BL/6 mice were obtained from Jackson Laboratory (Bar Harbor, ME). The experiment started when the mice reached the age of 20 month. A younger cohort was studied in parallel at the age of 6 month. The older mice were already overweight with a fat/lean ratio about 1:2 at baseline while the younger mice were normal with fat/lean ration about 1:3 (see below). All animals went through body composition analysis by NMR and grip strength test at baseline and were then randomly assigned to control and Kaletra groups with matching body composition and grip strength. Control animals were given a diet containing fat 30.1%, CHO 48.4%, Protein 21.4% and the Kaletra group had the same diet but mixed with Kaletra (1000 mg lopinavir per kg). Both diets were custom-prepared by Harlan Taklan (TD.09547 for control and TD.09615 for Kaletra diet, [www.harlan.com](http://www.harlan.com/)). Kaletra was purchased from Abbott Laboratories as lopinavir/ritonavir (200/50 mg) pills and submitted to Harlan Teklad, a manufacturer for laboratory rodent research diets. The pills were homogenized and extracted with oil (part of the high fat diet ingredient) and then mixed with other ingredients of the diet following the industry standard procedure for rodent diet preparation with customer supplied ingredients (details available from www.harlan.com). Daily food consumption by mice is about 10% of their body weight. Hence, a 30 gm mouse typically consumed about 3 mg lopinavir each day, giving a drug dosage close to 100 mg/kg/day. It has been shown that after normalization to body surface area, Kaletra dosage of 125 mg/kg/day in mice translates to clinical dosage of 10 mg/kg/day in humans [1]. Hence, the daily drug consumption of our mice is similar to the human dosage. Of note, drug clearance is much faster in mice than in humans. Some of the previous studies used relatively high dosage for oral gavage or injection in order to maintain serum drug concentration for a longer period of time. However, it has been shown that regardless of the amount of Kaletra dosed (by oral gavage), serum lopinavir concentration reaches zero after 8 hours [2] while in humans taking Kaletra (400/100 mg, BID), the drug remains relatively stable in the range of 1.2 – 7.1 ug/ml throughout 24 hours [3]. We decided to use food-based drug delivery approach in part to reduce the acute surge of serum drug concentration associated with oral gavage or injection and also to sustain a longer presence of drug at a more physiological concentration range in the body, to better mimic the drug conditions in human patients. This strategy is similar to a drinking water-based drug delivery protocol reported by several previous rodent studies to administer ritonavir and other HIV protease inhibitors [4-6].

1.2. **Serum drug concentration**

Lopinavir and ritonavir in serum was quantified by HPLC analysis using a protocol modified from that reported by Verbesselt et al. [7]. The HPLC system consisted of a Waters (Milford, MA) 600 E pump with a controller, a photodiode array detector 996 (Waters), a manual injector 717 plus with cooling module (Waters) and a chromatographic data management system (Millennium32, Waters). Analytes were separated isocratically on an Allsphere hexyl 5 u column (150mm×4.6mm i.d.) (Alltech Associates Inc., purchased through [www.fisherscientific.com](http://www.fisherscientific.com/)). The mobile phase was a mixture of acetonitrile, methanol and 15mM sodiumdihydrogenphosphate buffer pH 4.5 (35/20/45, v/v/v) and the flow rate was 0.4 ml/min. The UV detector was set at 215 nm. Pure lopinavir and ritonavir were obtained from NIH AIDS reagent program ([www.aidsreagent.org](http://www.aidsreagent.org/)) and used as the calibration standard. Lopinavir and ritonavir were extracted from serum of drug-treated animals following the protocol reported by Verbesselt et al. [7] without modification. Different amount of pure lopinavir (2 – 32 ug/ml) and ritonavir (25% of lopinavir, wt/wt) were added to control serum and extraction was performed in parallel. The peak areas obtained from the latter were plotted against known input drug concentrations to construct the standard curve for the unknown samples. All samples were analyzed in triplicates. The lopinavir concentrations measured from serum obtained at the time of sacrifice are 4.21 +/- 0.43 ug/ml for the older animals and 2.97 +/- 0.68 ug/ml for the younger animals. These results are within the normal range of serum drug concentration in humans (min 1.2 /max 7.14 ug/ml on lopinavir/ritonavir 400 mg/100 mg BID, Hsu et al., 2003) and mice (3.1 +/- 0.3 ug/ml, lopinavir/ritonavir 50/12.5 mg/kg, 4 h after i.p injection [1]).

1.3. **Gripping strength**

Gripping force is an indicator of muscle strength and the technique has been validated in many studies assessing muscular function under different patho-physiological conditions [8-10]. This method has also been shown as one of the most representative measurements of functional decline in both human and rodents [9,11-13]. Besides, a decline in handgrip strength is parallel with cognitive decline in older humans [14-18]. Since forelimb and hind limb grip force has been shown to be positively correlated in rodents [8], only forelimb gripping strength was tested in this study. For each animal, gripping strength was measured at baseline and at the end of the experiment. An automated gripping-strength transducer (Columbus Instrument, Columbus, OH) was used for this test following a standard protocol provided by Jackson Laboratory ([www.jax.org](http://www.jax.org/)).

1.4. **Rota-rod**

A 4-lane accelerating rota-rod (Columbus Instrument) was used for this test.  Each lane is equipped with a built-in automatic timer. The speed of the rotating rod is adjusted manually. Mice were trained first at low speed from 5 rpm and gradually increased by 1 rpm/min to 9 rpm (for the old) and 35 rpm (for the young) consecutively for 3 days. For the running test, mice were placed on a static rod. Once all animals were on the rod, the motor was turned on and the rod rotation was continuously accelerated at a rate of 0.5 rpm/min until all animals fell off the rod. The speed for each animal at the fall was recorded. The measurement was repeated three times on different days and the mean value was presented as the maximal achievable running speed.

1.5. **Clams**

Comprehensive Lab Animal Monitoring System (Columbus Instrument) was used to assess the effect of Kaletra on ambulatory respiration and activity of the mice following the instrument protocol as described in our previous work [19]. Five weeks after the initiation of drug treatment, animals were place in metabolic cages with free access to food and water for two days. Respiration and ambulatory motion were recorded automatically. To allow sufficient time for acclimation, only the data acquired on the second day were analyzed.

1.6. **Insulin and glucose tolerance tests**

Insulin and glucose tolerance tests were performed towards the end of the experiment (wk 6^th^ -7^th^). For insulin tolerance test, food was removed at 8 am and insulin (~100 ul, 0.6 U/kg, i.p. prepared in saline containing 0.01% bovine serum albumin) was injected at 12 pm. For glucose tolerance test, food was removed at 8 am. Glucose (~ 100 ul, i.p. 2 gm/kg, prepared in saline) was injected at 4 pm. Blood glucose was measured at baseline and every 15 min after injection of glucose or insulin. Results were plotted as blood glucose concentration as a function of time.

# 1.7. Cell culture and treatment

# C2C12 murine myoblasts were obtained from ATCC ([**www.atcc.org**](http://www.atcc.org/)) and grown in high glucose Dulbecco's Modified Eagle Medium (DMEM) containing 10% fetal bovine serum and standard antibiotics. To induce differentiation, fetal bovine serum was switched to 2% horse serum for four days. Lopinavir and ritonavir were obtained from NIH AIDS Research & Reference Reagent Program ([**www.aidsreagent.org**](http://www.aidsreagent.org/)). To determine the drug effect on IGF1 expression, myoblasts or mature myotubes were incubated for 24 h with graded concentration of lopinavir/ritonavir pre-dissolved in DMEM with 2% horse serum. Cells were harvested for RT-qPCR analysis of IGF1 and its related target genes. To determine the drug effect on IGF1 signaling, myotubes were incubated for 24 h with lopinavir/ritonavir (4:1) at 3 ug/ml of lopinavir in DMEM containing 2% horse serum. Cells were then washed with serum-free medium and incubated with serum-free medium with the same concentration of lopinavir/ritonavir for two additional hours. IGF1 was then added and cells were harvested after 30 mins and used for Western analysis. All standard cell culture supplies were purchased from Invitrogen ([**www.invitrogen.com**](http://www.invitrogen.com/)) or Fisher Scientific ([**www.fishersci.com**](http://www.fishersci.com/)).

1.8. **Tissue and plasma triglyceride concentrations**

Animals were euthanized at the end of 7 weeks of drug treatment. Tissue and plasma were harvested at the time of sacrifice between 10 am to 12 pm each day. Food was removed at 8 am on the day of sacrifice (rodents typically consumed their major meals shortly after dark and once more shortly before light, which correspond to 8 – 9 pm and 4 - 5 am, respectively). All tissue samples were weighed and immediately frozen in liquid nitrogen and stored at -80°C until use. Blood was drawn by heart puncture and mixed with EDTA. After centrifuged at 3000 g for 15 min, plasma was collected and stored at -80°C until use. Plasma samples were packed with dry ice and sent to the animal clinical laboratory at University of North Carolina at Chapel Hill (www.med.unc.edu/anclinic) for triglyceride analysis. For tissue triglyceride analysis, tissue samples were first lyophilized and lipids were extracted by Folch’s method. Triglyceride was analyzed using a commercial kit from Sigma.

1.9. **Tissue and cellular DNA, RNA, and protein analysis**

Total RNA was extracted from frozen tissues using RNeasy mini kit (Qiagen, Valencia, CA). For reverse transcription, 1 µg of the total RNA was converted to first-strand complementary DNA in 20 µl reactions using Affinity Script Q-PCR cDNA Synthesis Kit (Agilent Technologies, La Jolla,CA), which was subsequently diluted five times. Real-time PCR analysis was performed using ABI 7500 system as described before [20]. For PCR sample preparation, 5 µl of cDNA was mixed in 20 µl reaction volume with 10 µmol/l primer and SYBR master enzyme mix (SABiosciences, Frederick, MD). The reaction was initiated at 94°C for 10 min, followed by 40 cycles through 94°C for 15 s and 60°C for 1 min. All reactions were performed in duplicate. All CT values were in the range of 20-30 cycles. Amplification curves were analyzed using SDS 1.9.1 software (Applied Biosystems, Foster City, CA). House-keeping gene hypoxanthine phosphoribosyltransferase (HPRT) was used as endogenous control for estimation of relative gene expression levels. All PCR primers were intron-spanning designed using a web-based PCR primer design tool and shown in supplemental Table s1.

1.10. **Western analysis**

Western analysis was performed following the protocols described in our previous work [21]. All antibodies used for this work were purchased from Cell Signaling ([www.cellsignal.com](http://www.cellsignal.com/)).

1.11. **Statistics**

All data were presented as means +/- se. Comparison between control and Kaletra-treated groups were conducted using Student’s *t* test. Multiple group comparison was conducted by One Way ANOVA followed with post hoc Tukey’s test.

1.12. **Justification for food-based drug delivery**

Although many of the prior studies used oral gavage or ip injection for drug delivery, these approaches can cause acute surge of serum drug concentration as compared to the serum concentration achievable in human serum. In addition, drug clearance is faster in mice than in humans. Lopinavir concentration drops from 17 ug/ml to zero in 8 hours in a mouse after oral gavage of 400/100 mg/kg [2], while it remains in the range of 1.2 – 7.1 ug/ml throughout 24 h in humans taking 400/100 mg BID [3]. We speculate that daily acute surge in drug exposure followed with a prolonged drug-free period may cause repetitive impairment of adipogenic machinery [22] and also prevent endogenous adaptation, while constant exposure to drugs at lower concentration could do the opposite. In part for this consideration, we used a food-based drug delivery system. While the total daily drug intake was not very different from the previous rodent studies, our approach allowed extended periods for drug delivery, which is expected to reduce the acute drug surge and sustain a lower drug concentration for longer time throughout the day, to better mimic the serum and tissue drug exposure in human patients. Unfortunately, we do not have the 24 hour serum drug concentration data to attest this speculation, although we did found a moderate lopinavir concentration in the sera collected in the late morning (3 – 5 ug/ml), which is about 5 – 7 hours after the last major food intake for nocturnal animals. Nevertheless, the findings of drug-related abdominal fat enlargement from our study appear to be consistent with the clinical reports on patients taking Kaletra [23]. The impact of drug delivery protocol on metabolic and functional performance in preclinical animal models warrants further investigation.

**Supplemental table 1: Primers used for real Time Q-PCR**

|  | Forward primer | Reverse primer |
| --- | --- | --- |
| PPARγ2 | 5'-agctgcaggccattctacc-3' | 5'-tcaggggtagggtgtcagtc-3' |
| C/EBPα | 5'-aaacaacgcaacgtggaga-3' | 5'-gcggtcattgtcactggtc-3' |
| Adiponectin | 5'-ggagagaaaggagatgcaggt-3' | 5'-ctttcctgccaggggttc-3' |
| TNFα | 5'-tccgcttgctacttgactga-3' | 5'-gccagggcctcatactctc-3' |
| IGF-1Ea | 5'-gacatgcccaagaccagaagga-3' | 5'-cggtggcatgtcactcttcactc-3' |
| MuRF1 | 5'-tgacatctacaagcaggagtgc-3' | 5'-tcgtcttcgtgttccttgc-3' |
| MaFbx | 5'-agtgaggaccggctactgtg-3' | 5'-gatcaaacgcttgcgaatct-3' |
| MyoG | 5'-ccttgctcagctccctca-3' | 5'-tgggagttgcattcactgg-3' |
| CCND1 | 5'-tttctttccagagtcatcaagtgt-3' | 5'-tgactccagaagggcttcaa-3' |
| CCND3 | 5'-ggcatactggatgctggag-3' | 5'-ccaggtagttcatagccagagg-3' |

Note: PPARγ2 - *Peroxisome proliferator-activated receptor gamma 2,* C/EBPα - *Ccaat-enhancer-binding proteins,* TNFα - *Tumor necrosis factor-alpha,* IGF-1Ea - *Insulin-like growth factor-1isoforms Ea,* MuRF1 - *Muscle RING finger protein 1,* MaFbx – *Muscle Atrophy F-box/Atrogin1,* MyoG – *Myogenin,* CCND1- *cyclin D1,* CCND3 - *cyclin D3*

Supplemental Reference

1. [Pistell PJ, Gupta S, Knight AG, Domingue M, Uranga RM, et al. (2010) Metabolic and neurologic consequences of chronic lopinavir/ritonavir administration to C57BL/6 mice. Antiviral Res 88: 334-342.](http://www.ncbi.nlm.nih.gov/pubmed/20970459)
2. [Prot M, Heripret L, Cardot-Leccia N, Perrin C, Aouadi M, et al. (2006) Long-term treatment with lopinavir-ritonavir induces a reduction in peripheral adipose depots in mice. Antimicrob Agents Chemother 50: 3998-4004.](http://www.ncbi.nlm.nih.gov/pubmed/17000748)
3. [Hsu A, Isaacson J, Brun S, Bernstein B, Lam W, et al. (2003) Pharmacokinetic-pharmacodynamic analysis of lopinavir-ritonavir in combination with efavirenz and two nucleoside reverse transcriptase inhibitors in extensively pretreated human immunodeficiency virus-infected patients. Antimicrob Agents Chemother 47: 350-359.](http://www.ncbi.nlm.nih.gov/pubmed/12499212)
4. [Bradshaw EL, Li XA, Guerin T, Everson WV, Wilson ME, et al. (2006) Nucleoside reverse transcriptase inhibitors prevent HIV protease inhibitor-induced atherosclerosis by ubiquitination and degradation of protein kinase C. Am J Physiol Cell Physiol 291: C1271-1278.](http://www.ncbi.nlm.nih.gov/pubmed/16822947)
5. [Allred KF, Smart EJ, Wilson ME (2006) Estrogen receptor-alpha mediates gender differences in atherosclerosis induced by HIV protease inhibitors. J Biol Chem 281: 1419-1425.](http://www.ncbi.nlm.nih.gov/pubmed/16299001)
6. [Dressman J, Kincer J, Matveev SV, Guo L, Greenberg RN, et al. (2003) HIV protease inhibitors promote atherosclerotic lesion formation independent of dyslipidemia by increasing CD36-dependent cholesteryl ester accumulation in macrophages. J Clin Invest 111: 389-397.](http://www.ncbi.nlm.nih.gov/pubmed/12569165)
7. [Verbesselt R, Van Wijngaerden E, de Hoon J (2007) Simultaneous determination of 8 HIV protease inhibitors in human plasma by isocratic high-performance liquid chromatography with combined use of UV and fluorescence detection: amprenavir, indinavir, atazanavir, ritonavir, lopinavir, saquinavir, nelfinavir and M8-nelfinavir metabolite. J Chromatogr B Analyt Technol Biomed Life Sci 845: 51-60.](http://www.ncbi.nlm.nih.gov/pubmed/16997640)
8. [Kehl LJ, Trempe TM, Hargreaves KM (2000) A new animal model for assessing mechanisms and management of muscle hyperalgesia. Pain 85: 333-343.](http://www.ncbi.nlm.nih.gov/pubmed/10781907)
9. [Carter CS, Cesari M, Ambrosius WT, Hu N, Diz D, et al. (2004) Angiotensin-converting enzyme inhibition, body composition, and physical performance in aged rats. J Gerontol A Biol Sci Med Sci 59: 416-423.](http://www.ncbi.nlm.nih.gov/pubmed/15123750)
10. [Marzetti E, Leeuwenburgh C (2006) Skeletal muscle apoptosis, sarcopenia and frailty at old age. Exp Gerontol 41: 1234-1238.](http://www.ncbi.nlm.nih.gov/pubmed/17052879)
11. [Tatar M (2009) Can we develop genetically tractable models to assess healthspan (rather than life span) in animal models? J Gerontol A Biol Sci Med Sci 64: 161-163.](http://www.ncbi.nlm.nih.gov/pubmed/19225031)
12. [Kirkland JL, Peterson C (2009) Healthspan, translation, and new outcomes for animal studies of aging. J Gerontol A Biol Sci Med Sci 64: 209-212.](http://www.ncbi.nlm.nih.gov/pubmed/19196900)
13. [Xu J, Knutson MD, Carter CS, Leeuwenburgh C (2008) Iron accumulation with age, oxidative stress and functional decline. PLoS One 3: e2865.](http://www.ncbi.nlm.nih.gov/pubmed/18682742)
14. [Alfaro-Acha A, Al Snih S, Raji MA, Kuo YF, Markides KS, et al. (2006) Handgrip strength and cognitive decline in older Mexican Americans. J Gerontol A Biol Sci Med Sci 61: 859-865.](http://www.ncbi.nlm.nih.gov/pubmed/16912105)
15. [Anstey KJ, Smith GA (1999) Interrelationships among biological markers of aging, health, activity, acculturation, and cognitive performance in late adulthood. Psychol Aging 14: 605-618.](http://www.ncbi.nlm.nih.gov/pubmed/10632148)
16. [Nourhashémi F, Andrieu S, Gillette-Guyonnet S, Reynish E, Albarède JL, et al. (2002) Is there a relationship between fat-free soft tissue mass and low cognitive function? Results from a study of 7,105 women. J Am Geriatr Soc 50: 1796-1801.](http://www.ncbi.nlm.nih.gov/pubmed/12410897)
17. [Christensen H, Mackinnon AJ, Korten AE, Jorm AF, Henderson AS, et al. (1999) An analysis of diversity in the cognitive performance of elderly community dwellers: individual differences in change scores as a function of age. Psychol Aging 14: 365-379.](http://www.ncbi.nlm.nih.gov/pubmed/10509693)
18. [MacDonald SW, Dixon RA, Cohen AL, Hazlitt JE (2004) Biological age and 12-year cognitive change in older adults: findings from the Victoria Longitudinal Study. Gerontology 50: 64-81.](http://www.ncbi.nlm.nih.gov/pubmed/14963373)
19. [Tu P, Bhasin S, Hruz PW, Herbst KL, Castellani LW, et al. (2009) Genetic disruption of myostatin reduces the development of proatherogenic dyslipidemia and atherogenic lesions in Ldlr null mice. Diabetes 58: 1739-1748.](http://www.ncbi.nlm.nih.gov/pubmed/19509018)
20. [Guo W, Jiang L, Bhasin S, Khan SM, Swerdlow RH (2009) DNA extraction procedures meaningfully influence qPCR-based mtDNA copy number determination. Mitochondrion 9: 261-265.](http://www.ncbi.nlm.nih.gov/pubmed/19324101)
21. [Guo W, Wong S, Pudney J, Jasuja R, Hua N, et al. (2009) Acipimox, an inhibitor of lipolysis, attenuates atherogenesis in LDLR-null mice treated with HIV protease inhibitor ritonavir. Arterioscler Thromb Vasc Biol 29: 2028-2032.](http://www.ncbi.nlm.nih.gov/pubmed/19762785)
22. [Vernochet C, Azoulay S, Duval D, Guedj R, Ailhaud G, et al. (2003) Differential effect of HIV protease inhibitors on adipogenesis: intracellular ritonavir is not sufficient to inhibit differentiation. AIDS 17: 2177-2180.](http://www.ncbi.nlm.nih.gov/pubmed/14523274)
23. [Shah K, Hilton TN, Myers L, Pinto JF, Luque AE, et al. (2012) A new frailty syndrome: central obesity and frailty in older adults with the human immunodeficiency virus. J Am Geriatr Soc 60: 545-549.](http://www.ncbi.nlm.nih.gov/pubmed/22315957)
